# CircSEC24A (hsa_circ_0003528) interference suppresses epithelial-mesenchymal transition of hepatocellular carcinoma cells via miR-421/MMP3 axis

**DOI:** 10.1080/21655979.2022.2057761

**Published:** 2022-04-10

**Authors:** Bo Zhang, Jian Zhou

**Affiliations:** a Department of Basic Medicine, Chongqing Medical and Pharmaceutical College, Chongqing, China; b Department of Pathology, The Affiliated Hospital of Southwest Medical University, Luzhou, Sichuan, China

**Keywords:** hepatocellular carcinoma (HCC), circSEC24A, hsa_circ_0003528, miR-421, MMP3, competing endogenous RNA

## Abstract

Accumulating evidence indicates that circular RNAs (circRNAs) function as conclusive modulators in diverse tumors, including in hepatocellular carcinoma (HCC). Nonetheless, knowledge of the latent mechanisms involving circRNAs in HCC development is insufficient. circSEC24A (hsa_circ_0003528) was discovered by microarray analysis of patients with HCC. Binding sites between circSEC24A, miR-421, miR-421 and matrix metalloproteinase 3 (MMP3) were predicted using online bioinformatics tools. Interactions involving miRNA and target genes or circRNAs were verified by luciferase reporter-gene and RNA pull-down assays. Two HCC cell lines (HCCLM3 and Hep3B) and normal THLE-2 liver cells were used for *in vitro* experiments. miRNA and mRNA expression levels were detected by RT-qPCR, and protein expression was measured by western blotting. Cell proliferation was evaluated using Cell Counting Kit 8 (CCK-8) assays along with colony formation assays. Cell invasion and migration were determined using the Transwell and wound healing migration assays. A xenograft model was used to evaluate the role of circSEC24A *in vivo*. circSEC24A expression was significantly upregulated in HCCLM3 and Hep3B cells. Silencing circSEC24A mitigated the proliferation, migration, invasion, and epithelial-mesenchymal transition (EMT) of HCC cells, which was abrogated by downregulation of miR-421. Meanwhile, MMP3 could bind to miR-421 to decrease the functional effects of miR-421 and induce tumor metastasis. Knockdown of cicSEC24A suppressed tumor growth *in vivo*. circSEC24A interference suppressed HCC cell EMT by sponging miR-421, further regulating MMP3, and inhibiting tumor growth *in vivo*. Therefore, circSEC24A could represent a potential target for HCC patient treatment.

## GraphicalAbstract

**Figure:**
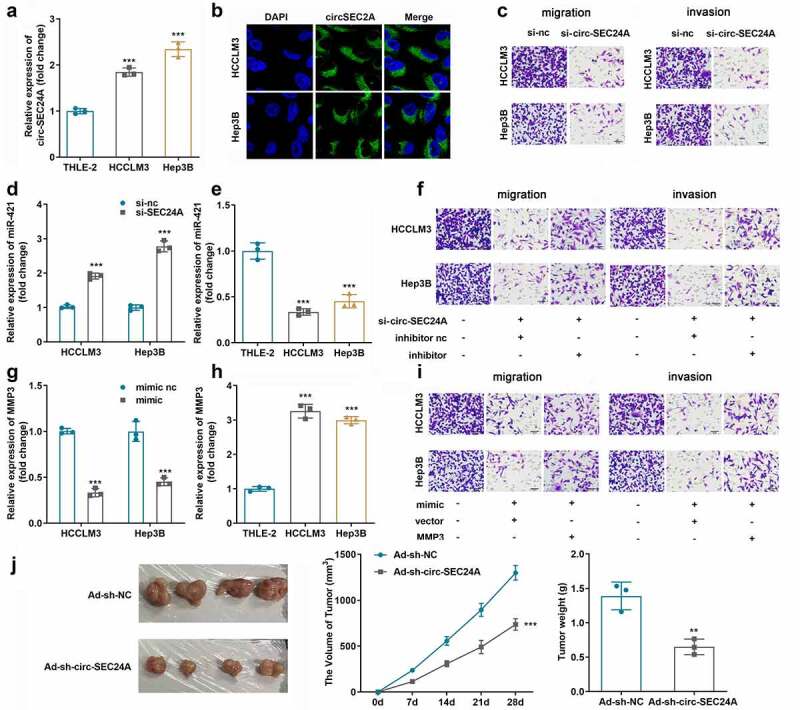

## Introduction

Hepatocellular carcinoma (HCC) is one of the most widely recognized cancers, with high incidence, mortality, and recurrence [1–3]. It has an insidious onset at an early stage, and there remains a lack of effective and reliable early screening [1–3]. Over the past three decades, despite significant advances in surgery and other treatment techniques, the five-year survival rate for HCC is still very poor, resulting in severe economic losses and a heavy social burden [4]. The initiation and progression of HCC involves multiple genes and environmental factors. Multiple pathological stages and changes involving various molecular events are required to realize the evolution of normal hepatocytes into HCC cells and even for eventual metastasis [5,6]. Therefore, it is vital to explore the underlying molecular mechanisms.

Previous studies have shown that epithelial-to-mesenchymal transition (EMT) is associated with cell polarity and cellular motor abilities. EMT is involved in the regulation of malignant biological behaviors of tumors [7,8]. The most significant molecular event during EMT is a decrease in E-cadherin expression and augmentation of N-cadherin and vimentin expression [9,10]. Recent studies have uncovered the fundamental function of EMT in HCC metastasis [11,12], highlighting the need to further investigate the molecular mechanisms involved in EMT in HCC.

Covalently closed circular RNAs (circRNAs) are non-coding RNAs connected by the back-splicing of exons (3' and 5' ends) or introns of precursor mRNAs, which are ubiquitously expressed in eukaryotes [13]. Recently, various studies have identified circRNAs as diagnostic and therapeutic targets for modulating tumor progression and metastasis [14,15]. In HCC, several circRNAs have been clarified to be involved in HCC progression, e.g. circ-SOX5 [16], circZFR [17]. Different from circRNAs's structure, miRNAs are another cluster of small non-coding RNAs which have a length of ~22 nucleotides [18], which considered to be important mediators in many biological process involved in HCC progression [19]. More importantly, alterations affecting circRNA expression have been reported to be linked to EMT to modulate tumor metastatic behavior [20,21]. circSEC24A is a newfound circRNA that has been reported to accelerate tumor progress in squamous cell carcinoma [22] and pancreatic cancer [23]. However, the function of circSEC24A in HCC is indistinct.

In this study, the expression profile of circRNAs in patients with HCC was investigated, and circSEC24A (circBase ID: hsa_circ_0003528) was found to be abnormally expressed in HCC using bioinformatics tools. Our in-depth study demonstrated that circSEC24A features HCC metastasis through the miR-421–MMP3 axis *in vitro* and *in vivo*.

## Materials and methods

### Microarray analysis

The GSE94508 dataset obtained from the GEO database was employed to select for differential expression analysis in HCC patients. The online software tool GEO2R was applied to the filtrate of dysregulated circRNAs [24]. |fold change| ≥ 2 and *p* < 0.05 were set as the criteria to identify significant differentially expressed circRNAs. Then, we identified the gene source of circRNA in circBase (http://www.circbase.org/) according to the sequence of the most significantly up-regulated circrRNA to determine the name of the circRNA. Additionally, predicted binding sites between circRNA, miRNA, and target genes were performed by the online tool Starbase v.3.0 (https://starbase.sysu.edu.cn/) and TargetScan v.7.2 (http://www.targetscan.org/mmu_72/).

### Patients

In total, 37 pairs of HCC and adjacent normal tissues were surgically removed from patients undergoing primary HCC surgery in the Affiliated Hospital of Southwest Medical University. Written informed consent was obtained from all patients, and the study was approved by the ethics committee and was conducted in accordance with the Declaration of Helsinki.

### Cell lines

Two HCC cell lines (HCCLM3 and Hep3B) and normal THLE-2 hepatocytes were purchased from Shanghai Cell Bank, Chinese Academy of Sciences. HCC cell lines were maintained in DMEM (Thermo Fisher Scientific, CA, USA), while normal hepatocytes were cultured in RPMI-1640 medium (Thermo Fisher Scientific) supplemented with 10% fetal bovine serum (FBS; Jiangsu Ningyan Biomedical Research Institute Co. Ltd, Jiangsu, China) in a 5% CO_2_ incubator at 37°C.

### Fluorescence in situ hybridization (Fish)

A total of 10^4^ HCC cells were planted in 96-well plates. After the cells adhered to the wall, 4% paraformaldehyde was added to fix the cells (15 min), followed by 0.1% BufferA (15 min), Buffer C (30 min), 70% ethanol (3 min), 85% ethanol (3 min), and anhydrous ethanol (3 min). The probe was diluted with Buffer E, and incubated at 73°C for 5 min. After cooling, 100 μl of probe dilution was added to each well, and hybridized overnight at 37°C. Then Buffer F and Buffer C were successively added, DAPI working solution was added to avoid light staining for 20 minutes, and the location of circRNA in cells was determined under fluorescence microscope.

### Oligonucleotide transfection

All small interfering RNAs (siRNAs), including si-circSEC24A#1, si-circSEC24A#2, corresponding negative control (si-NC), miR-421 mimic (mimic) and corresponding control (mimic NC), miR-421 inhibitor (inhibitor) and corresponding control (inhibitor NC), MMP3 overexpression vector (MMP3), and corresponding control (vector) were obtained from VectorBuilder Biotech (Jiangsu, China). Cell transfection was performed using Lipofectamine 3000 [25] (Invitrogen, Carlsbad, CA, USA) in accordance with the manufacturer’s specifications until confluency reached 60%. After 24 h, the transfection efficiency was evaluated by quantitative reverse transcription polymerase chain reaction (RT-qPCR).

### RT-qPCR assay

mRNA and miRNA expression levels were determined by RT-qPCR, and all PCRs were performed in triplicate. TRIzol reagent (Beyotime Biotech, Jiangsu, China) was used to extract RNA from HCCLM3, Hep3B, and THLE-2 cells. RNA integrity and concentration were assessed using a spectrophotometer (NanoDrop Technologies, Thermo Fisher Scientific) as reported [26]. The quantified RNA was then reverse-transcribed using the SuperScript IV CellsDirect cDNA kit (Thermo Fisher Scientific). Quantitative polymerase chain reaction was carried out on an ABI StepOnePlus Real-Time PCR System/Applied Biosystems 7500 Real-Time PCR system (Thermo Fisher Scientific). All primers were synthesized by Nanjing GenScript Biotech (Jiangsu, China) and were as follows in Table 1. The expression levels of circSEC24A and MMP3 were normalized to that of *GAPDH*, and *U6* was used as an endogenous reference for miR-421 abundance.

**Table 1.** Primer sequence

Primer	Forward	Reverse
circSEC24A	5'-GCTCTCCTTAAACAGGATATACACAA-3'	5'-TGTCCACTGAGAAGGAATAAGTCA-3'
miR-421	5'-GTCGCGCGGGUUAAUGCCTC-3'	5'-GGACATUAGUUGUCUGUAAATAG-3'
MMP3	5'-AGTCTTCCAATCCTACTGTTGCT-3'	5'-TCCCCGTCACCTCCAATCC-3'
GAPDH	5'-AATGGACAACTGGTCGTGGAC-3'	5'-CCCTCCAGGGGATCTGTTTG-3'
U6	5'-AAAGCAAATCATCGGACGACC-3'	5'-GTACAACACATTGTTTCCTCGGA-3'

### Cell proliferation assays

Cell Counting Kit 8 (CCK-8; Beyotime Biotech) assays were used to measure cell viability from OD_450_ absorbance readings (Spectra Max 250 spectrophotometer; Molecular Devices, California, USA). After exponential phase, cells were seeded into 96-well plates, 10 μl CCK-8 reagent was added to each well at 6, 24, 48, 72, and 96 h [27]. After 1.5 h of incubation, absorbance was measured using a microplate reader. Each experiment was performed independently in triplicate.

Colony formation assays were performed to determine the colony numbers. Briefly, HCC cells were seeded into 6-well plates and maintained for 14 days. Emergent cell colonies were then fixed and incubated with 0.1% crystal violet and 20% methanol solution for 10 min. Visible colonies were photographed and counted using a microscope (Nikon, Tokyo, Japan). All experiments were performed at least thrice.

### Transwell assay

Cell migration and invasion abilities were measured using Transwell assays [28]. Cells (2 × 10^4^ cells/well) and 200 μl serum-free medium were added to the upper chamber of 24-well Transwell chambers (Corning, New York, USA). Medium (600 μl) containing 2% FBS was added to the bottom chambers to chemically attract the upper cells. The cells were incubated for 24 h in migration assays, and 48 h in invasion assays with Matrigel (BD Bioscience, California, USA). After the cells migrated or invaded through the membranes, they were stained with 0.5% crystal violet. The number of migrated or invaded cells was counted under a 200 × microscope in three random fields.

### Wound healing migration assay

Wound healing migration assay was used to assess the migration ability of HCC cells, 6 orifice cells covered by more than 90%, pipetting spear with straightedge perpendicular scratches inside the cell culture plate, each hole through the five line, scratches with PBS rinse cells after three times, cleared cross out the adherent cells, add transfection of cells after serum free medium, watch pictures after 0 h, After incubation at 37°C in a 5% CO_2_ incubator for 24 h, samples were taken again for observation and photographs were taken under an inverted fluorescence microscope.

### Western blot assay

The protein levels in HCC cells were detected by western blot assays, and primary antibodies were used for this assay, including anti-N-cadherin (ab76011) and anti-E-cadherin (ab40772) (1/10,000, Abcam, USA). The cells were collected and lysed in RIPA buffer (Beyotime Biotech). The BCA Protein Assay kit (Beyotime Biotech) was used to measure protein concentrations [29]. Proteins were separated by 12% SDS-PAGE and transferred onto nitrocellulose membranes (Biosharp, Anhui, China) after electrophoretic resolution. Subsequently, the membranes were sealed in 5% nonfat milk powder with TBS and 0.01% Tween and incubated with primary antibodies at 4°C overnight. Next, the membrane was incubated with secondary antibody for 1 h. Finally, an enhanced chemiluminescence kit (Beyotime Biotech) was used to visualize protein bands.

### Luciferase reporter-gene assay

HCCLM3 and Hep3B cells were seeded in 24-well plates overnight until cell confluence reached approximately 60%. Wild-type and mutant circSEC24A luciferase reporter-gene constructs containing the binding site for miR-421 were cloned into pGL3-control vector (Promega, Madison, WI, USA). Then, 250 ng of luciferase reporter was transfected into HCCLM3 and Hep3B cells with miR-421 mimic using Lipofectamine 3000 (Invitrogen). After transfecting for 48 h, firefly and Renilla luciferase fluorescence were measured using the Dual-Luciferase Reporter Assay kit (Promega). The binding relationship involving MMP3 and miR-421 was studied in a similar way [30].

### RNA pull-down assay

RNA pull-down assays were performed as previously described [31]. HCC cells were lysed and incubated with a biotinylated miR-421 probe and a negative control. Streptavidin-labeled magnetic beads (RNA Pull Down Assay Kit; 297–77,501; Whatman, UK) were added and cultured overnight at 4°C. RNA-protein complexes were eluted, resolved by SDS-PAGE, and stained with Silver Stain Plus (Beyotime Biotech). The results were determined by RT-qPCR.

### Xenograft animal model assay

Twelve BALB/c nude mice (male, 4-weeks-old) were purchased from Shanghai SLAC Laboratory Animal Center (Shanghai, China). Animal experiments met the demands of laboratory animal welfare and ethics. Hep3B cells transfected with sh-NC or sh-circSEC24A suspended in DMEM at 2 × 10^6^ were injected into the backs of mice and allowed to grow for 25 days to establish the xenograft model. 7–10 days after inoculation, the maximum and minimum diameters of the tumor were measured with vernal calipers twice a week, and tumor volume was estimated (volume = maximum and minimum diameters ^2^/2).

### Statistical analysis

All experiments were conducted thrice. GraphPad Prism 8.3 (GraphPad, CA, USA) and SPSS 19.0 (IBM, New York, USA) were used for data analysis. All data are shown as means ± SD. Differences were analyzed using Student’s *t*-test and one-way analysis of variance (ANOVA). Statistical significance was set at *p* < 0.05.

## Results

### Identification of aberrant expression of circSEC24A

**Figure 1.**
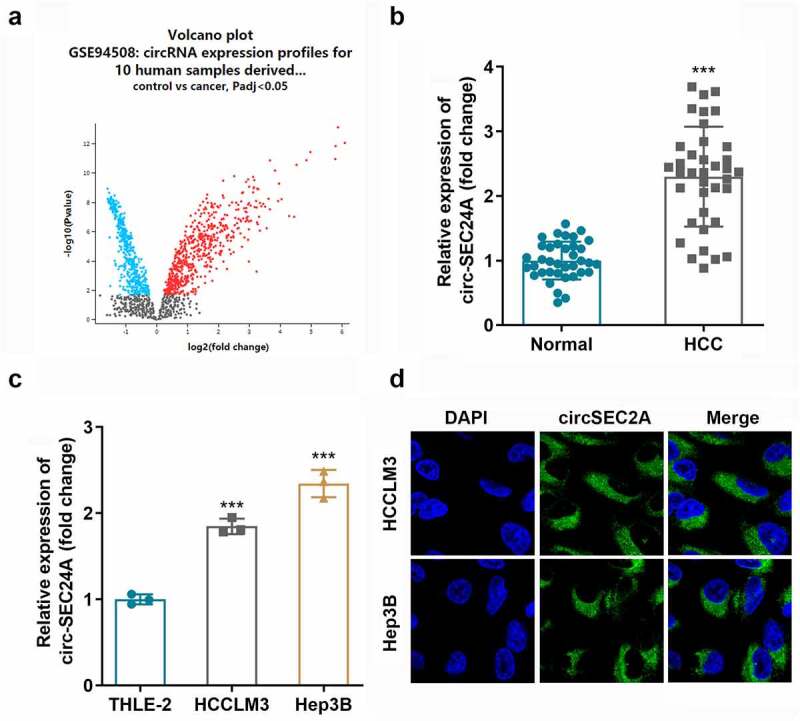
**Aberrant expression level of circSEC24A in HCC. A** Differentially expressed circRNAs in HCC clinical samples analyzed using an online dataset. B Expression of circSEC24A in HCC tissues and paracancer tissues measured by RT-qPCR. CExpression of circSEC24A in HCC cells measured by RT-qPCR. D The location of circSEC24A in HCC cells accessed by FISH method. ***p < 0.001.

We summarized differentially expressed circRNAs (DECs) obtained from the GSE94508 microarray dataset. As shown in the volcano map, 560 DECs were identified, including 214 up-regulated and 346 down-regulated circRNAs (Figure 1a). circRNA hsa_circ_0003528 (circSEC24A) was found to be the most significantly upregulated in HCC samples compared to that in controls (Figure 1b), which was verified in cell experiments. circSEC24A was markedly overexpressed in HCCLM3 and Hep3B cells (Figure 1c). Additionally, circSEC24A was found to be localized in the cytoplasm of HCC cells (Figure 1d).

### Silencing circSEC24A inhibited HCC cell proliferation, invasion, migration and EMT

**Figure 2.**
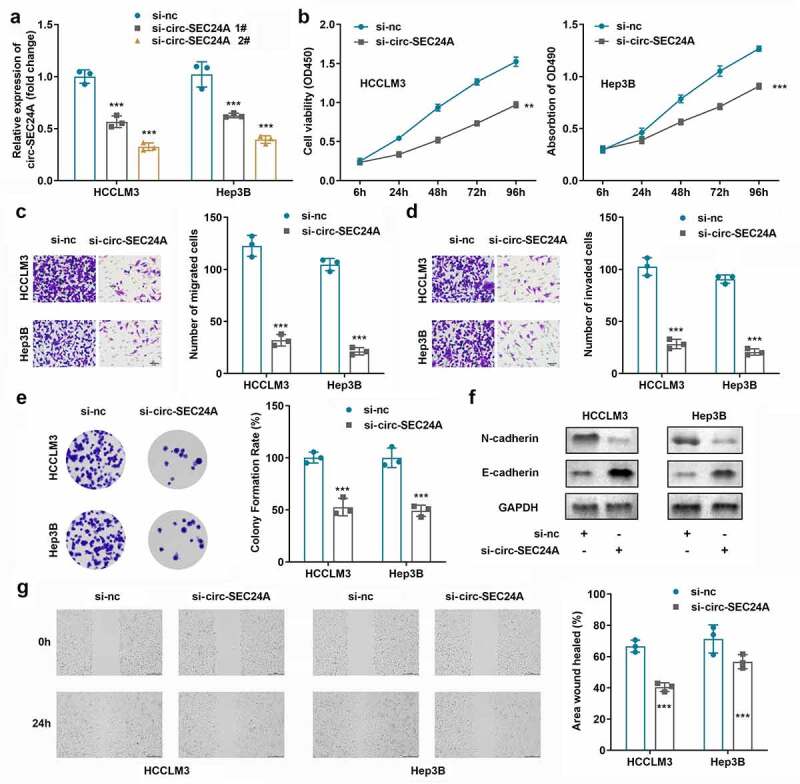
**Suppressed circSEC24A cellular functions on HCC cells**. A CircSEC24A expression was detected after transfection with interference plasmids. B Cell viability was detected using CCK8 assays. C-D. Cell migration and invasion abilities were assessed using Transwell assays. E. Colony formation assays were applied to detect cell proliferation. F. Expression of EMT-related proteins was detected by western blot. G. Wound healing migration assay was performed to measure cell migration **p < 0.01, ***p < 0.001, vs. si-NC group.

Then, whether circSE24A can regulate aggressive behaviors were invsteigated after knocking down of circSE24A. si-circSEC24A plasmids were transfected into HCCLM3 and Hep3B cells to suppress the expression of circSEC24A, demonstrating that circSEC24A was decreased more extensively after transfection with si-circSEC24A 2# plasmid (Figure 2a). The proliferation, invasion, and migration of both HCCLM3 and Hep3B cells were accessed by CCK-8, colony formation, transwell, scratch test methods, and the results indicated that downregulation of circSEC24A significantly inhibited the proliferation,invasion, and migration of HCCLM3 and Hep3B cells compared to those in the control group (Figure 2b-e, g). Meanwhile, western blot analysis demonstrated that N-cadherin protein was downregulated, while E-cadherin protein was upregulated by silencing circSEC24A (figure 2f).

### CircSEC24A functioned as a sponge for miR-421

**Figure 3.**
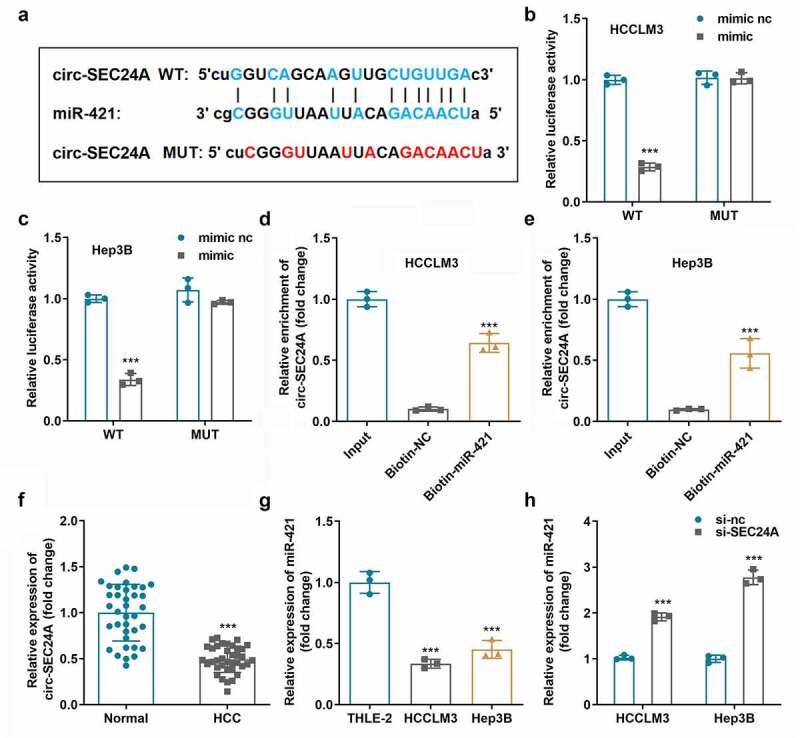
**miR-421 bound circSEC24A. A** Binding sites involving miR-421 and circSEC24A predicted by bioinformatic analysis. B-C. Luciferase reporter assays were conducted to confirm interactions between miR-421 and circSEC24A. D-E. Interaction between miR-421 and circSEC24A was verified using RNA pull-down assays. F. miR-421 expression in HCC tissues and paracancer tissues measured by RT-qPCR. G. miR-421 expression in HCC cells measured by RT-qPCR. H. miR-421 expression was measured after circSEC24A knockdown . ***p < 0.001, vs. mimic NC, si-NC, Biotin-NC, THLE-2 and normal group.

miR-421 was predicted to be a target of circSEC24A using the online tool Starbase v.3.0; the binding sites are illustrated in Figure 3a. Luciferase activity of both HCCLM3 and Hep3B cells was dramatically suppressed in cells transfected with the miR-421 mimic and wild-type circSEC24A (Figure 3(b,c)). RNA pull-down assays demonstrated that circSEC24A could be pulled-down by biotinylated miR-421 (Figure 3(d,e)). Moreover, the expression level of miR-421 appeared to be suppressed in HCC tissues (figure 3f) and two HCC cell lines (Figure 3g) tested relative to normal groups. Additionally, the expression level of miR-421 was significantly upregulated when circSEC24A expression was suppressed (Figure 3h).

### CircSEC24A may accelerate aggressiveness of HCC progression by regulating miR-421

**Figure 4.**
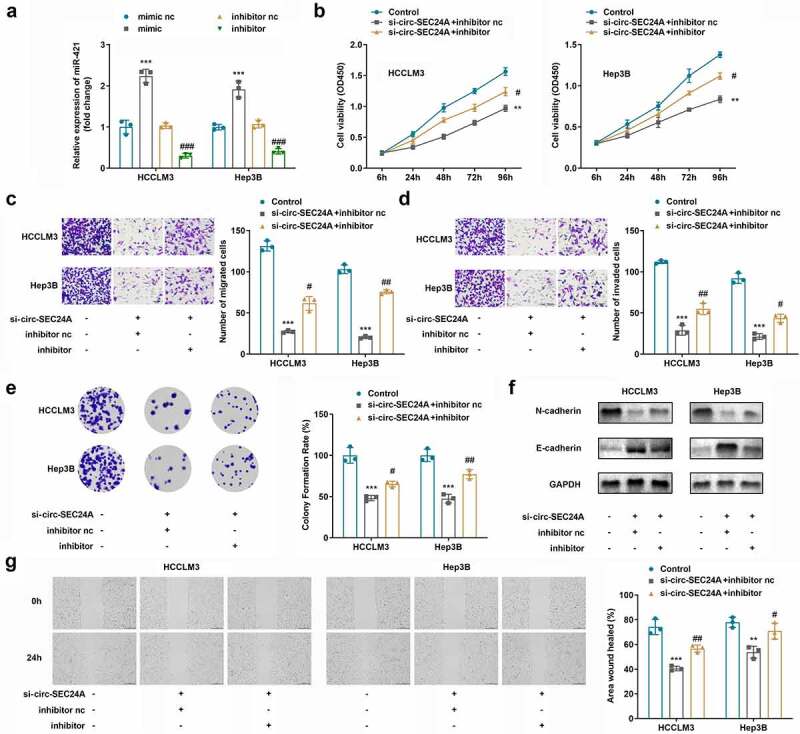
**Effects of miR-421 inhibitor on HCC cells. A**. miR-421 expression levels were detected by RT-qPCR. B Cell viability was determined using CCK8 assays. C-D. Cell migration and invasion abilities were assessed using Transwell assays. E. Colony formation assays were applied to detect cell proliferation. F. Expression of EMT-related proteins were detected by western blotting. G. Wound healing migration assay was performed to measure cell migration. **p < 0.01, ***p < 0.001, vs. mimic NC, control group. #*p* < 0.05, ##*p* < 0.01, ###*p* < 0.001, vs. inhibitor NC, si-circSEC24A + inhibitor group.

Next, we accessed aggressiveness of HCC cells by inhibiting miR-421. As indicated in Figure 4a, miR-421 expression was sharply suppressed in the miR-421 inhibitor group and increased in the miR-421 mimic cohort in both HCCLM3 and Hep3B cells. Downregulation of miR-421 partially abrogated the effects of circSEC24A on the proliferation, migration, invasion, and EMT of HCC cells (Figure 4b-g).

### MMP3 as a target of miR-421

**Figure 5.**
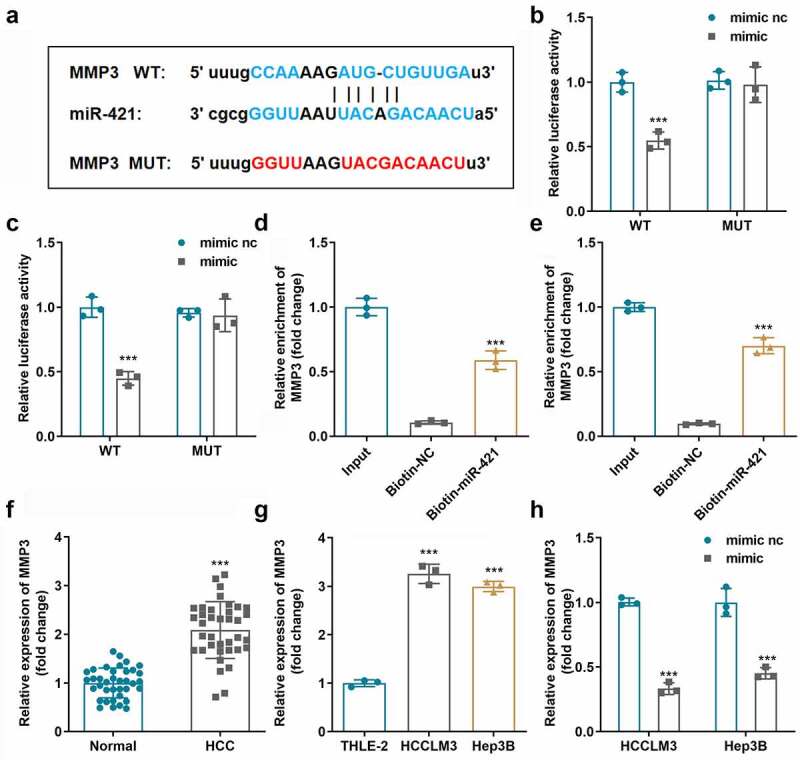
**MMP3 as a miR-421 target gene. A** Bioinformatic analysis predicted binding sites involving miR-421 and MMP3 B-C. Luciferase reporter-gene assays were conducted to confirm interactions between miR-421 and MMP3. D-E. Interaction between miR-421 and MMP3 was verified using RNA pull-down assays. F. MMP3 expression in HCC tissues and paracancer tissues measured by RT-qPCR. G. MMP3 expression in HCC cells measured by RT-qPCR. H. MMP3 expression was measured after miR-421 up-regulation. ***p < 0.001, vs. mimic NC, si-NC, Biotin-NC, THLE-2 group.

MMP3 is a target gene of miR-421 according to the online tool TargetScan v.7.2, and its binding sites are illustrated in Figure 5a. The results of luciferase and RNA pull-down assays confirmed the binding relationship involving MMP3 and miR-421 (Figure 5b-e). The expression of MMP3 mRNA was notably increased in HCC tissues and cells compared to that in normal groups (Figure 5(f,g)). Moreover, mRNA expression of MMP3 was prominently reduced by the miR-421 mimic (Figure 5h).

### Over-expressed MMP3 inhibited the effects of miR-421

**Figure 6.**
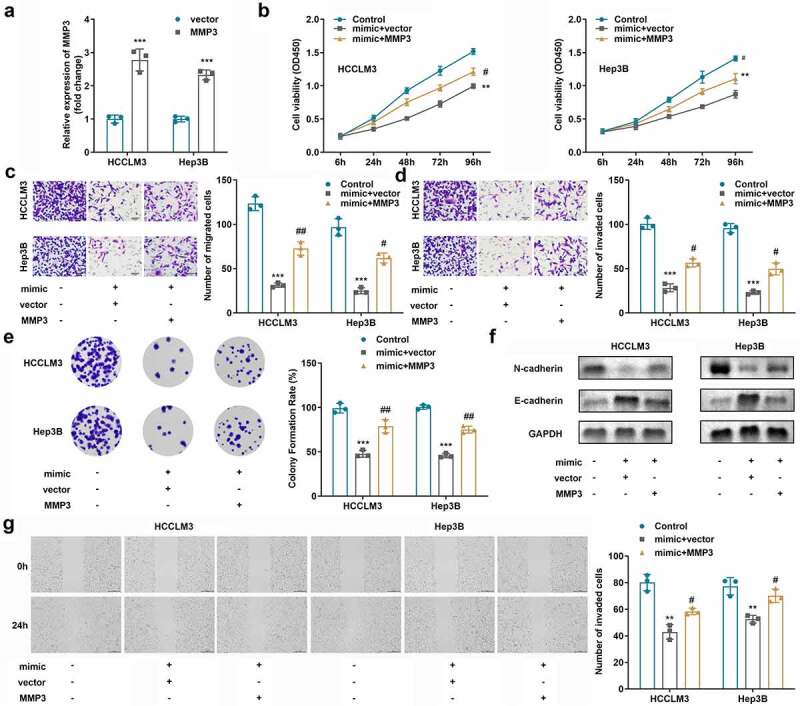
**Elevated MMP3 levels mitigated the effects of miR-421**. A. MMP3 expression was detected by RT-qPCR. b Cell viability was detected using CCK8 assays. C-D. Cell migration and invasion were evaluated using Transwell assays. E. Colony formation assays were applied to detect cell proliferation. F. Expression of EMT-related proteins was detected by western blotting. G. Wound healing migration assay was performed to measure cell migration. **p < 0.01, ***p < 0.001, vs. vector, control group. #*p* < 0.05, ##*p* < 0.01, vs. mimic+vector group.

To investigate the effects of MMP3 on miR-421, we overexpressed MMP3 by transfecting OE-MMP3 plasmid into HCCLM3 and Hep3B cells. As revealed in Figure 6a, co-transfection with miR-421 overexpression plasmid and the OE-MMP3 vector accelerated cell proliferation (Figure 6 b and e), migration (Figure (6 c,g)), invasion (Figure 6d), and the EMT process (figure 6f) in HCC cells. Furthermore, upregulated MMP3 partly antagonized the cellular functions of miR-421 in HCC cells.

### Depletion of circSEC24A inhibited tumor growth in vivo

**Figure 7.**
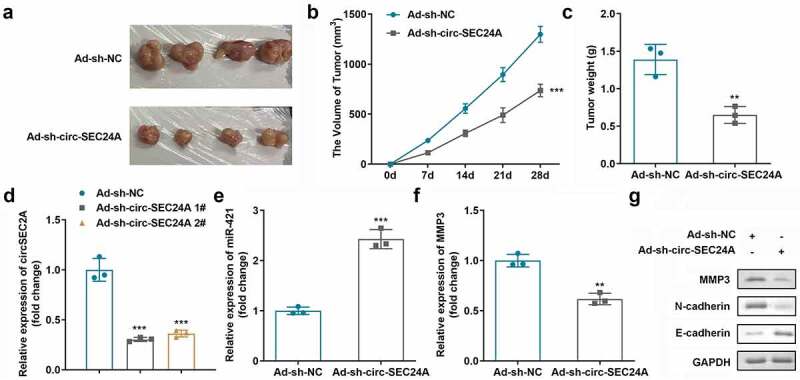
**Effects of sh-circSEC24A on xenograft model *in vivo***. Representative images (a), tumor volumes (b), and tumor weights (c) of tumors derived from xenograft animal models. RT-qPCR analyses of (d) circSEC24A, (e) miR-421, and (f) MMP3 expression. (g) EMT related proteins were measured by western blotting assay. **p < 0.01, ***p < 0.001, vs. Ad-sh-NC group.

To elucidate the function of circSEC24A in HCC, a xenograft tumor model was established, and tumor volumes and weights were analyzed. As revealed in Figure 7d, two sh-circSEC24A plasmids obviously suppressed circSEC24A expression *in vivo*, which was more potent in 1# group (Figure 7d). What’s more, miR-421 could also be elevated while MMP was suppressed *in vivo* by inhibition of circSEC24A (Figure 7(e,f)). Additionally, the results showed that tumor volume (Figure 7a-b) and weight (Figure 7c) in the sh-circSEC24A group were significantly lower than those in the control group. Furthermore, protein levels of MMP3 and N-cadherin in tumor tissues were suppressed while E-cadherin was upregulated (Figure 7g).

## Discussion

In this study, circSEC24A was abnormally elevated in HCC, and knockdown of circSEC24A inhibited cell proliferation, invasion, migration, and EMT progression in *in vitro* functional assays. Our findings revealed that circSEC24A competes with MMP3 for miR-421 binding, thereby manipulating cell fate and promoting tumorigenesis.

Dysregulation of circRNAs, identified using RNA sequencing technologies along with online bioinformatic analysis has been reported to regulate the initiation and development of diverse disorders including HCC [15,32,33]. Numerous studies have suggested that certain circRNAs influence the occurrence and development of HCC. For instance, over-expression of circRNA Cdr1as facilitates aggressive behavior of HCC cells by modulating miR-1270 [33]. Yao et al. [34] demonstrated that circ_0001955 expedites HCC carcinogenesis through competing endogenous RNA (ceRNA) mechanisms. Our study identified the potential role of circSEC24A in HCC. Functional assays indicated that silencing circSEC24A retarded HCC development by inhibiting the proliferation, migration, invasion, and EMT of HCC cells. *In vivo* experiments further showed that knockdown of circSEC24A suppressed tumor growth. Hence, circSEC24A has been postulated to act as an oncogene in HCC.

Emerging evidence has shown that circRNAs can act as ceRNAs and isolate specific miRNAs from target genes, thereby inhibiting miRNA stability and function [32,35]. Our data indicated that circSEC24A might bind to miR-421, which is known to play a crucial role in tumors. For instance, miR-421 serves as an oncogene in osteosarcoma [36], ovarian cancer [37], glioma [38], pancreatic cancer [39], ovarian cancer [40], and HCC [41], whereas a small fraction of the data show that miR-421 plays an oncogenic role in HCC [42]. Therefore, it is essential to identify the regulatory mechanism involving miR-421 in HCC. Our study demonstrated that miR-421 levels were decreased in HCC cells, which is in accord with previous research results [41]. Moreover, inhibition of miR-421 abrogated the suppressive functional effects of silencing circSEC24A in HCC, indicating that circSEC24A downregulation inhibited the aggressiveness of HCC cells by upregulating miR-421.

Our data suggested that MMP3 binds to miR-421. MMP3 is a member of the MMP family, and alterations to MMPs *in vivo* play crucial roles in promoting tumor invasion and metastasis [43,44]. MMP3 has been reported to promote tumor invasion and metastasis in a variety of tumors [45–47], including HCC [48]. Wang et al. [48] showed that miR-30a-3p suppresses tumor formation by reducing the expression of MMP3 in HCC cells. Likewise, downregulation of miR-17 dramatically suppresses cell migration and invasion *in vitro* and *in vivo* by inhibiting MMP3 [49]. These studies demonstrate that MMP3 may play a positive role in tumor cell invasion and migration. In this study, MMP3 levels were elevated in HCC cells, and overexpression of MMP3 abrogated the effect of miR-421 on cell proliferation, invasion, and migration inhibition. Furthermore, EMT progression by cells was suppressed by upregulated MMP3, which is in accord with previous studies [50,51]. Scheau et al. [52] reported that MMP3 participates in the EMT process in HCC and plays a role in promoting tumor invasion and metastasis, which is consistent with the findings of this study.

## Conclusion

Collectively, these results suggest that circSEC24A interference suppresses HCC development by regulating the miR-421/MMP3 axis. Therefore, circSEC24A could represent a potential target for HCC patient treatment.
